# Ixr1 Is Required for the Expression of the Ribonucleotide Reductase Rnr1 and Maintenance of dNTP Pools

**DOI:** 10.1371/journal.pgen.1002061

**Published:** 2011-05-05

**Authors:** Olga Tsaponina, Emad Barsoum, Stefan U. Åström, Andrei Chabes

**Affiliations:** 1 Department of Medical Biochemistry and Biophysics, Umeå University, Umeå, Sweden; 2 Department of Developmental Biology, Wennergren Institute, Stockholm University, Stockholm, Sweden; 3 Laboratory for Molecular Infection Medicine Sweden (MIMS), Umeå University, Umeå, Sweden; University of Washington, United States of America

## Abstract

The *Saccharomyces cerevisiae* Dun1 protein kinase is a downstream target of the conserved Mec1-Rad53 checkpoint pathway. Dun1 regulates dNTP pools during an unperturbed cell cycle and after DNA damage by modulating the activity of ribonucleotide reductase (RNR) by multiple mechanisms, including phosphorylation of RNR inhibitors Sml1 and Dif1. Dun1 also activates DNA-damage-inducible genes by inhibiting the Crt1 transcriptional repressor. Among the genes repressed by Crt1 are three out of four RNR genes: *RNR2*, *RNR3*, and *RNR4*. The fourth RNR gene, *RNR1*, is also DNA damage-inducible, but is not controlled by Crt1. It has been shown that the deletion of *DUN1* is synthetic lethal with the deletion of *IXR1*, encoding an HMG-box-containing DNA binding protein, but the reason for this lethality is not known. Here we demonstrate that the *dun1 ixr1* synthetic lethality is caused by an inadequate RNR activity. The deletion of *IXR1* results in decreased dNTP levels due to a reduced *RNR1* expression. The *ixr1* single mutants compensate for the reduced Rnr1 levels by the Mec1-Rad53-Dun1-Crt1–dependent elevation of Rnr3 and Rnr4 levels and downregulation of Sml1 levels, explaining why *DUN1* is indispensible in *ixr1* mutants. The *dun1 ixr1* synthetic lethality is rescued by an artificial elevation of the dNTP pools. We show that Ixr1 is phosphorylated at several residues and that Ser366, a residue important for the interaction of HMG boxes with DNA, is required for Ixr1 phosphorylation. Ixr1 interacts with DNA at multiple loci, including the *RNR1* promoter. Ixr1 levels are decreased in Rad53-deficient cells, which are known to have excessive histone levels. A reduction of the histone gene dosage in the *rad53* mutant restores Ixr1 levels. Our results demonstrate that Ixr1, but not Dun1, is required for the proper *RNR1* expression both during an unperturbed cell cycle and after DNA damage.

## Author Summary

Dun1 is a non-essential protein kinase important for the maintenance of genome stability in budding yeast. Earlier studies found that simultaneous deletion of *DUN1* and *IXR1* results in lethality, but the reason for this so-called synthetic lethality is not clear. Ixr1 is implicated in DNA repair based on its ability to bind to DNA modified by the anticancer drug cisplatin. Here, we investigated the mechanism behind the *ixr1 dun1* synthetic lethality. We demonstrate that yeast strains lacking Ixr1 have decreased amounts of dNTPs, the building blocks of DNA. This is because Ixr1 is required for the normal expression of Rnr1, one of the essential subunits of the enzyme ribonucleotide reductase (RNR), which catalyzes the rate-limiting step in the production of all four dNTPs. Cells lacking Ixr1 compensate the decreased expression of Rnr1 by the increased expression of other *RNR* genes and degradation of RNR inhibitors. These compensatory processes require Dun1. Hence, cells lacking both Dun1 and Ixr1 have dNTP pools that are too low for survival. Our work identifies a new important player in the synthesis of the building blocks of DNA.

## Introduction

Cells experiencing DNA damage or replication blocks activate stress response pathways, or checkpoints, that arrest the cell cycle and facilitate DNA repair. In budding yeast, the key checkpoint protein kinases are Mec1 (homolog of human ATR) and Rad53 (homolog of CHK2 and functional homolog of CHK1 in human), reviewed in [1], [2]. In human cells, ATR and CHK2 are upstream regulators of p53 and are inactivated in many cancers.

**Figure 1:**
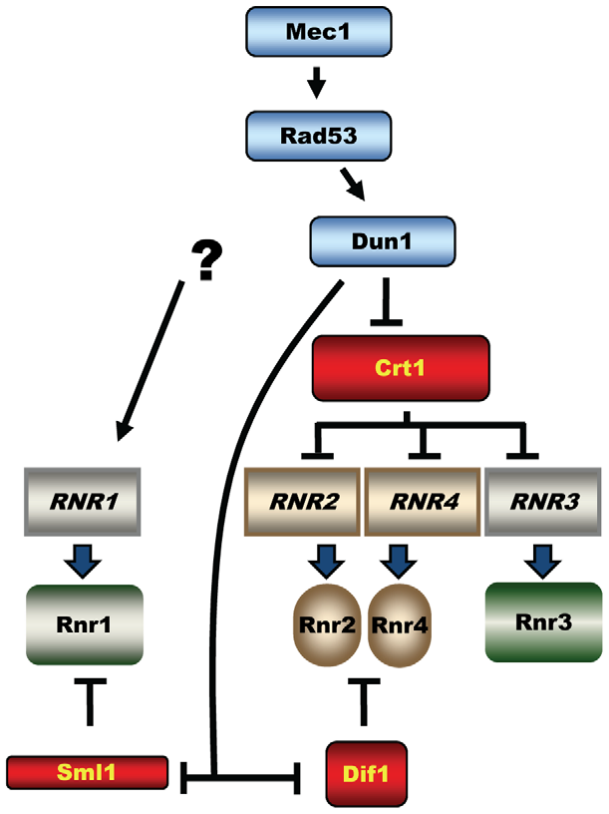
Mec1-Rad53-Dun1–dependent regulation of *S. cerevisiae* ribonucleotide reductase. The activated Dun1 kinase relieves inhibition of RNR by targeting the transcriptional repressor Crt1(Rfx1) and protein inhibitors Sml1 and Dif1.

In *Saccharomyces cerevisiae*, Mec1 and Rad53 are essential and control phosphorylation and activation of the checkpoint kinase Dun1 [3] (Figure 1). Dun1 shares homology with Rad53 and Chk2 and is required for the DNA damage response. The essential function of the Mec1-Rad53-Dun1 pathway is to maintain an adequate supply of dNTPs by regulating the activity of ribonucleotide reductase (RNR) during the normal cell cycle [4]–[6]. RNR catalyzes the rate-limiting step in the biosynthesis of all four dNTPs and maintains both their balance and appropriate concentration. Full activation of RNR in *S. cerevisiae* by the Mec1-Rad53-Dun1 checkpoint in response to DNA damage results in a 6- to 8-fold increase in dNTP concentration [7]. Such increases in dNTP concentration during DNA damage correlate with DNA damage tolerance. Four genes encode yeast RNR: *RNR1* and *RNR3* encode the large subunit [8], [9], and *RNR2* and *RNR4* encode the small subunit [10]–[13].

The three key targets of the Mec1-Rad53-Dun1 pathway are Sml1, a protein inhibitor of RNR; Crt1 (Rfx1), a transcription factor; and Dif1, a protein that regulates the nuclear retention of Rnr2 and Rnr4 (Figure 1). Phosphorylation of Sml1 during S phase or after DNA damage by Dun1 targets Sml1 for proteolysis, which relieves the inhibition of RNR activity [14]. Phosphorylation of Dif1 releases Rnr2 and Rnr4 into the cytoplasm, where they combine with Rnr1 to form an active RNR complex [15], [16]. Crt1 blocks transcription at target promoters through recruitment of the general repressors Tup1 and Ssn6 [17]. Phosphorylation of Crt1 in a Mec1-Rad53-Dun1–dependent manner after DNA damage or replication stress promotes its dissociation from target promoters and activation of transcription. Crt1 represses *RNR2*, *RNR3*, and *RNR4* [12], [17]. *RNR3* is not essential and is normally expressed at very low levels, but is highly induced by DNA damage and has been used in genetic screens for the identification of both *DUN1* and *CRT1* [3], [17]. Interestingly, the fourth RNR gene, *RNR1*, is also DNA damage inducible but does not contain the Crt1-binding sites in its promoter and consequently is not repressed by Crt1. The mechanism of *RNR1* activation by DNA damage remains unknown [17], [18].

The lethality of *mec1* and *rad53* mutants can be rescued either by deletion of *SML1* (suppressor of mec1 lethality) [5], *CRT1* [17], *DIF1* [15], [16], or by overexpression of *RNR1* or *RNR3* [6], all resulting in increased RNR activity. In contrast, the deletion of *DUN1* is not lethal and does not cause any obvious proliferation defects except for a slightly prolonged S phase, defects in mitochondrial propagation and decreased dNTP levels [3], [14], [19]. It is therefore possible that another pathway exists downstream of Mec1 and Rad53, functioning in parallel with Dun1. Alternatively, the elevation of dNTP levels by the Mec1-Rad53-Dun1 pathway is essential only in *mec1* or *rad53* mutants, because Mec1 and Rad53, in contrast to Dun1, are involved in a plethora of important chromosomal transactions, reviewed in [1].

Others have performed large-scale analyses of synthetic genetic interactions, in which the *DUN1* gene was one of the baits. In two of such screens, *DUN1* mutants were found to be synthetic lethal with a gene encoding the intrastrand cross-link recognition protein (Ixr1), but the reason for this synthetic interaction remains unknown [20], [21]. Ixr1 is a high mobility group (HMG) transcription factor first identified by its ability to bind DNA modified by the anticancer drug cisplatin (cis-diamminedichloriplatinum(II)) [22]. Very little is known about the cellular function of Ixr1. In addition to the two HMG boxes, Ixr1 has several polyglutamine regions, important for protein–protein interactions. Its closest homolog in yeast is Abf2 (TFAM in human), a mitochondrial DNA-binding protein important for replication and transcription [23]. Earlier, Ixr1 was implicated in aerobic transcriptional repression of *COX5b*, which encodes a subunit of mitochondrial cytochrome c oxidase [24].

Here we demonstrate that Ixr1 is required for the maintenance of the Rnr1 levels. In the absence or Ixr1, Rnr1 levels are decreased and became even lower after DNA damage, instead of increasing as in the wild-type. This observation explains the sensitivity of *ixr1* mutants to hydroxyurea (HU), an inhibitor of RNR. In contrast, the levels of Rnr3 and Rnr4 in *ixr1* mutants are increased due to the activation of the Mec1-Rad53-Dun1-Crt1 pathway, and increase even further after DNA damage and replication blocks, similar to wild-type. We show that deletion of *SML1* or overexpression of *RNR1* or *RNR3* elevates dNTP pools and rescues the *ixr1 dun1* synthetic lethality. The requirement for RNR activation in *ixr1* via Dun1 explains why Dun1 is indispensible in *ixr1* mutants.

## Results

### *dun1*Δ is synthetic lethal with *ixr1-S366F*

**Figure 2:**
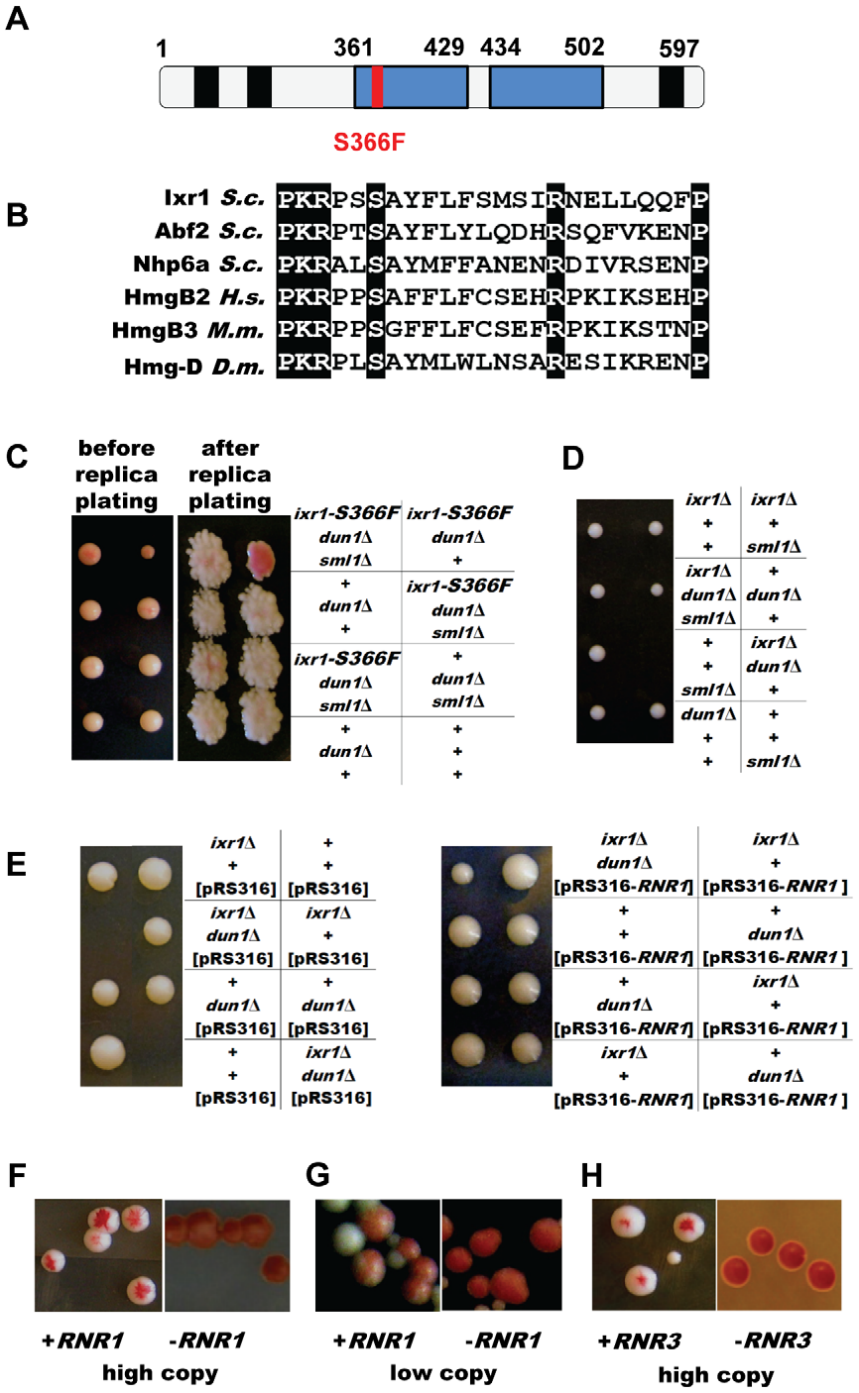
*dun1 ixr1* synthetic lethality is rescued by increased RNR activity. (A) Schematic representation of the Ixr1 protein. HMG boxes are shown in blue and polyQ regions in black. (B) Alignment of homologous HMG proteins close to the conserved S366. (C) Tetrad analysis demonstrating that deletion of *SML1* rescues the *dun1*Δ *ixr1-S366F* synthetic lethality. The *dun1*Δ *ixr1-S366F* strain (TOY544) carries a *DUN1*-containing plasmid, pK503, which confers a red color; this plasmid is lost in *ixr1-S366F dun1*Δ *sml1*Δ colonies (TOY544×TOY588). (D) Tetrad analysis demonstrating that deletion of *SML1* rescues the *ixr1*Δ *dun1*Δ synthetic lethality (TOY604×TOY527). (E) Tetrad analysis demonstrating that a low-copy *RNR1* vector rescues the *ixr1*Δ *dun1*Δ synthetic lethality. The diploid strain *ixr1/IXR1 dun1/DUN1* (TOY527×TOY603) was transformed with pRS316 and pBJ6 (pRS316-*RNR1*), sporulated and tetrad analysis was performed. (F) Overexpression of *RNR1* rescues the *ixr1-S366F dun1*Δ synthetic lethality. The pK503 plasmid, containing the *DUN1* gene and conferring red color, is lost on YP-Gal medium in the *ixr1-S366F dun1*Δ strain transformed with pESC-URA-pGAL1-*RNR1* plasmid (left panel, sectoring phenotype), but not when transformed with pESC-URA-pGAL1 (right panel). (G) Low-copy *RNR1* vector rescues the *ixr1-S366F dun1*Δ synthetic lethality. The pK503 plasmid, containing the *DUN1* gene and conferring red color, is lost in the *ixr1-S366F dun1*Δ strain transformed with the pBJ6 plasmid (left panel, sectoring phenotype), but not when transformed with pRS316 (right panel). (H) Overexpression of *RNR3* rescues the *ixr1-S366F dun1*Δ synthetic lethality. The pK503 plasmid, containing the *DUN1* gene and conferring red color, is lost in the *ixr1-S366F dun1*Δ strain transformed with pBAD79 plasmid (left panel, sectoring phenotype), but not when transformed with pRS414 (right panel).

Earlier screens for synthetic genetic interactions between *dun1*Δ and other genes used a collection of yeast strains with null alleles in all nonessential genes. To facilitate identification of synthetic genetic interactions of *DUN1* with essential genes we performed a colour-based synthetic lethal screen using a *dun1*Δ strain as described in the Materials and Methods. Briefly, the *ade2 ade3* yeast strains are white unless a plasmid with *ADE3* is present, conferring red color. Three mutations resulting in synthetic lethality with *dun1*Δ (first designated as *mut1*, *mut2*, and *mut3*) were isolated based on the inability of the *ade2 ade3 dun1*Δ *mut* strains to lose the pK503 plasmid carrying *DUN1* and *ADE3*. *MUT1* was identified as *RAD53*, *MUT2* as *WHI3*, and *MUT3* as *IXR1*. Sequencing of the *rad53* mutant allele identified a single point mutation changing His 622 to Tyr. The H622 residue of Rad53 was identified before as crucial for the interaction of Rad53 with Rad9 [25], but the synthetic lethality of *rad53-H622Y* was not known. Sequencing of *whi3* identified a single point mutation changing Gln in position 481 to a stop codon. Consistent with this observation, deletion of *WHI3* showed a synthetic growth defect with *dun1*Δ in a large-scale analysis [20]. Finally, sequencing of the *ixr1* mutant allele identified a single point mutation that changed Ser 366 to Phe in the first of the protein's two HMG boxes (Figure 2A). This highly conserved serine residue (Figure 2B) forms water-mediated hydrogen bonds to DNA bases and interacts with DNA [26]. In this study, we concentrated our efforts on the genetic interaction of *DUN1* with *IXR1*.

### Deletion of *SML1* or elevated expression of RNR genes rescues *dun1*Δ *ixr1* synthetic lethality

A well-established role of Dun1 is to increase RNR activity by targeting Sml1 and Dif1 for degradation and by transcriptional activation of *RNR2*, *RNR3* and *RNR4*. Therefore, we asked whether deletion of *SML1* or elevated expression of *RNR* genes rescues the lethality of *dun1*Δ *ixr1*. The *dun1*Δ *ixr1-S366F* [pK503] strain, originally identified in the screen, was crossed with a *dun1*Δ *sml1*Δ strain. Both strains are *ade2 ade3* mutants. The unstable pK503 plasmid was lost in *dun1*Δ *sml1*Δ *ixr1-S366F* colonies, based on their white color, but not in *dun1*Δ *ixr1-S366F* colonies (Figure 2C). Next, we crossed *ixr1*Δ *sml1*Δ with *dun1*Δ. Tetrad analysis confirmed that *dun1*Δ *ixr1*Δ *sml1*Δ spores were viable while *dun1*Δ *ixr1*Δ were unable to germinate (Figure 2D). An additional copy of *RNR1* rescues the *dun1 ixr1* synthetic lethality, as demonstrated by sporulation and tetrad analysis of the *ixr1/IXR1 dun1/DUN1* diploid strain transformed with a centromeric plasmid pBJ6 containing *RNR1* under the control of the native promoter (Figure 2E). Finally, after transformation with plasmids overexpressing *RNR1* (Figure 2F) or *RNR3* (Figure 2H) or with the centromeric pBJ6 plasmid containing an additional copy of *RNR1* (Figure 2G), we also observed the loss of the pK503 plasmid from the *dun1*Δ *ixr1-S366F* strain, leading to the sectoring phenotype.

### Rnr3 and Rnr4 levels are increased in *ixr1* mutants, while Sml1 levels are decreased

**Figure 3:**
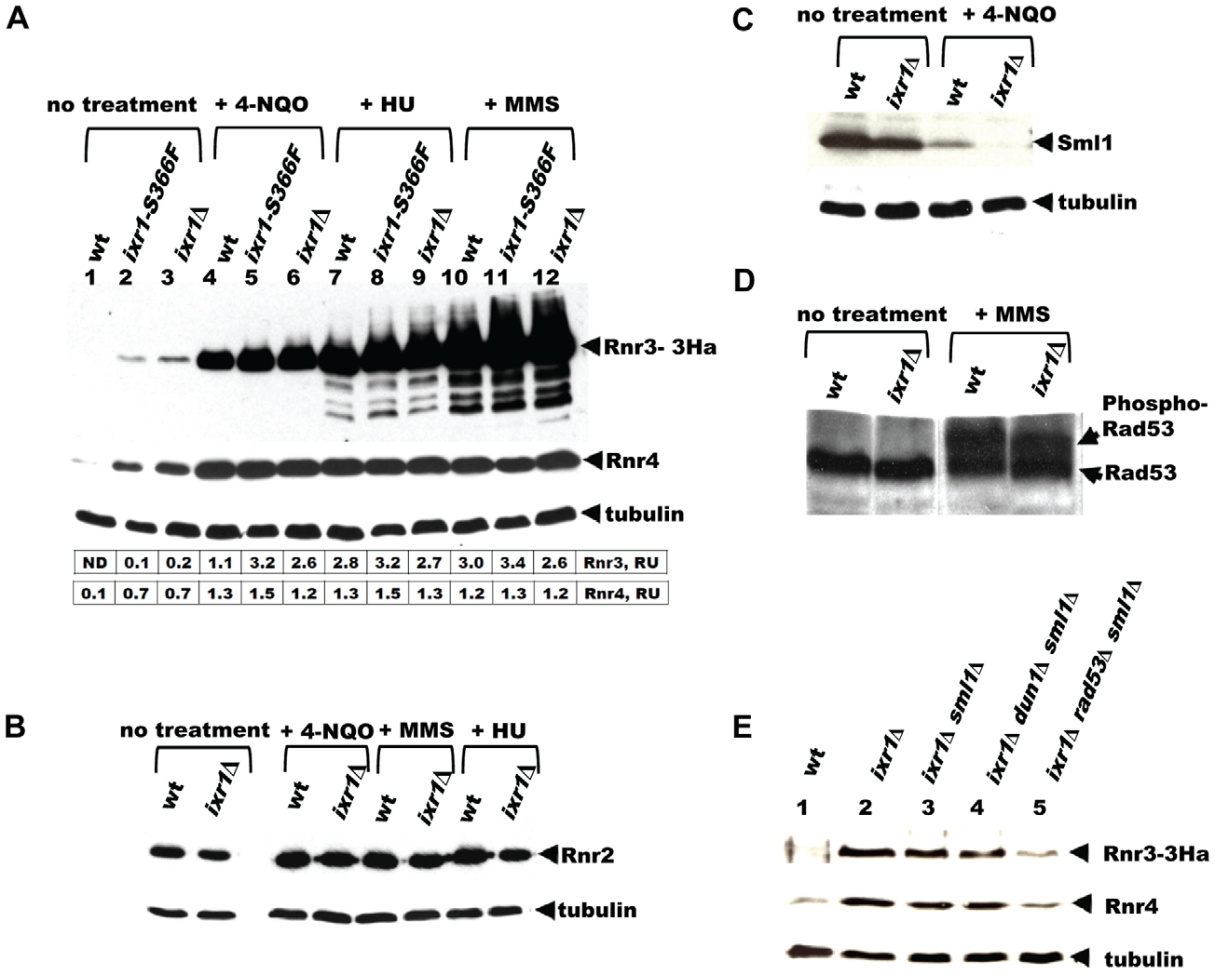
Deletion of *IXR1* leads to increased Rnr3 and Rnr4 levels and decreased Sml1 levels. (A) Western blot analysis of Rnr3-HA and Rnr4 levels in wild-type (AC447-2A), *ixr1-S366F* (TOY619), and *ixr1*Δ (TOY621) strains before and after 2 hours treatment with 0.2 mg/L 4-nitroquinoline 1-oxide (4-NQO), 200 mM HU, or 0.02% methyl methanesulfonate (MMS). Rnr3 and Rnr4 levels were quantified in relative units (RU, levels of Rnr3 or Rnr4 divided by the levels of tubulin in corresponding sample) as described in Materials and Methods. ND – not detected. (B) Western blot analysis of Rnr2 levels in wild-type (AC447-2A) and *ixr1*Δ (TOY621) strains before treatment and after 2 hours treatment with 0.2 mg/L 4-NQO, 0.02% MMS, or 200 mM HU. (C) Western blot analysis of Sml1 levels in wild-type (W1588-4C) and *ixr1*Δ (TOY736) strains before treatment and after 2 hours treatment with 0.02% MMS. (D) Western blot analysis of Rad53 phosphorylation status in wild-type (W1588-4C) and *ixr1*Δ (TOY736) strains before treatment and after 2 hours treatment with 0.02% MMS. (E) Deletion of *RAD53* but not of *SML1* or *DUN1* abolishes the upregulation of Rnr3 and Rnr4 levels in *ixr1*Δ. Western blot analysis of Rnr3-HA and Rnr4 levels. The following strains were analyzed: wt (AC447-2A), *ixr1*Δ (TOY732), *ixr1*Δ *sml1*Δ (TOY778), *ixr1*Δ *dun1*Δ *sml1*Δ (TOY772), and *ixr1*Δ *rad53*Δ *sml1*Δ (TOY781).

The requirement of Dun1 for *ixr1* viability suggests that the Mec1-Rad53-Dun1 pathway is activated in *ixr1* strains. A highly sensitive readout of this pathway's activation is induction of Crt1-controlled *RNR2*, *RNR3*, and *RNR4*. Indeed, Rnr3 and Rnr4 levels were higher in *ixr1-S366F* and *ixr1*Δ strains compared to wild-type (Figure 3A, lanes 1–3). Rnr2 levels were not significantly changed in *ixr1*Δ compared to wild-type (Figure 3B). Activation of the Mec1-Rad53-Dun1 pathway in *ixr1* strains was not maximal; exposure to DNA damaging agents further increased Rnr2, Rnr3 and Rnr4 levels (Figure 3A and 3B). We were able to detect an increase in Rnr3-HA levels in the undamaged *ixr1*Δ strain only with the more sensitive anti-HA antibodies, but not with the polyclonal anti-Rnr3 antibodies, also indicating that the Mec1-Rad53-Dun1 pathway activation is low (Figure S1A). Importantly, the elevation of Rnr2, Rnr3 and Rnr4 in response to DNA damage was identical in the *ixr1* and wild-type strains, indicating that the Mec1-Rad53-Dun1 pathway was not compromised in *ixr1* mutants. Sml1 levels were decreased in *ixr1*, again suggesting that the Mec1-Rad53-Dun1 pathway was activated (Figure 3C). We did not observe a mobility shift of the Rad53 band in *ixr1*Δ, indicating low activation of the Mec1-Rad53-Dun1 pathway (Figure 3D). The full activation of the checkpoint by DNA damage resulted in hyperphosphorylation of Rad53 both in *ixr1*Δ and wild-type, leading to a shift of the Rad53 band (Figure 3D).

Nevertheless, the increased Rnr3 and Rnr4 levels in *ixr1*Δ are Rad53 dependent. Deletion of *RAD53*, but not of *DUN1*, abolished upregulation of Rnr3 and Rnr4 in the *ixr1*Δ strain (Figure 3E). We interpret this result to mean that Rad53 can take over the Dun1 function and activate Rnr3 and Rnr4 expression in *ixr1*Δ when *DUN1* is deleted, but not *vice versa* because Dun1 functions downstream of Rad53. A *DUN1*-independent pathway for RNR transcriptional induction was observed earlier, and it was suggested that Rad53 can directly recognize Dun1 substrates [12]. Alternatively, Rad53, and not Dun1, could be the main activator of Rnr3 and Rnr4 expression in the *ixr1*Δ strain in the absence of DNA damage.

### dNTP levels are lower in the *ixr1*Δ strain compared to wild-type

**Figure 4:**
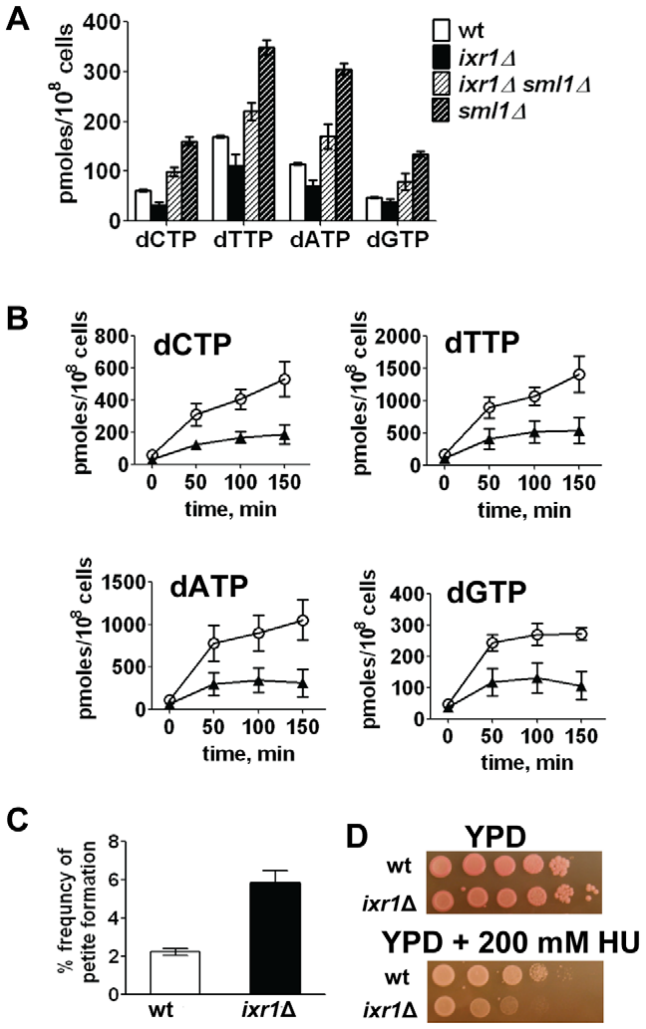
Deletion of *IXR1* leads to decreased dNTP levels. (A) dNTP levels are decreased in *ixr1*Δ (TOY736), increased in *ixr1*Δ *sml1*Δ (TOY714) compared to wild-type (W1588-4C), but lower than in *sml1*Δ (U952-3B). Values shown are the average from two independent experiments with the minimum and maximum values represented as error bars. (B) dNTP levels increase in *ixr1*Δ (TOY736, black triangles) during the treatment with 0.2 mg/L 4-NQO, but less than in a wild-type strain (W1588-4C, open circles). Values shown are the average from two independent experiments with the minimum and maximum values represented as error bars. (C) *ixr1*Δ (TOY736) has a higher frequency of petite formation than a wild-type strain (W1588-4C). (D) *ixr1*Δ (TOY736) is more sensitive to HU than a wild-type strain (W1588-4C).

Because the levels of several RNR proteins are increased (Rnr3, Rnr4) or unchanged (Rnr2) in *ixr1*Δ, while Sml1 levels are decreased, the dNTP levels might have been elevated in the *ixr1* strains; however, dNTP levels were lower in *ixr1*Δ compared to wild-type (Figure 4A). Similarly, dNTP levels in *ixr1*Δ *sml1*Δ were lower than in *sml1*Δ alone (Figure 4A). DNA damage induction by 4-nitroquinoline 1-oxide (4-NQO) resulted in an increase in dNTP concentration in *ixr1*Δ, but to a lower level compared to wild-type (Figure 4B). The *ixr1*Δ mutant exhibited an increased frequency of petite formation (Figure 4C) and sensitivity to HU (Figure 4D), two phenotypes associated with decreased dNTP production [5]. Interestingly, although the dNTP levels were higher in *ixr1*Δ *sml1*Δ than in wild type, Rnr3 and Rnr4 in *ixr1*Δ *sml1*Δ were still elevated to the same levels as in the *ixr1*Δ single mutant (Figure 3E). This observation indicates that activation of the Mec1-Rad53-Dun1 pathway in *ixr1*Δ is not only due to a decreased dNTP production, but also due to some other defects.

### Levels of Rnr1 in *ixr1* decrease after DNA damage

**Figure 5:**
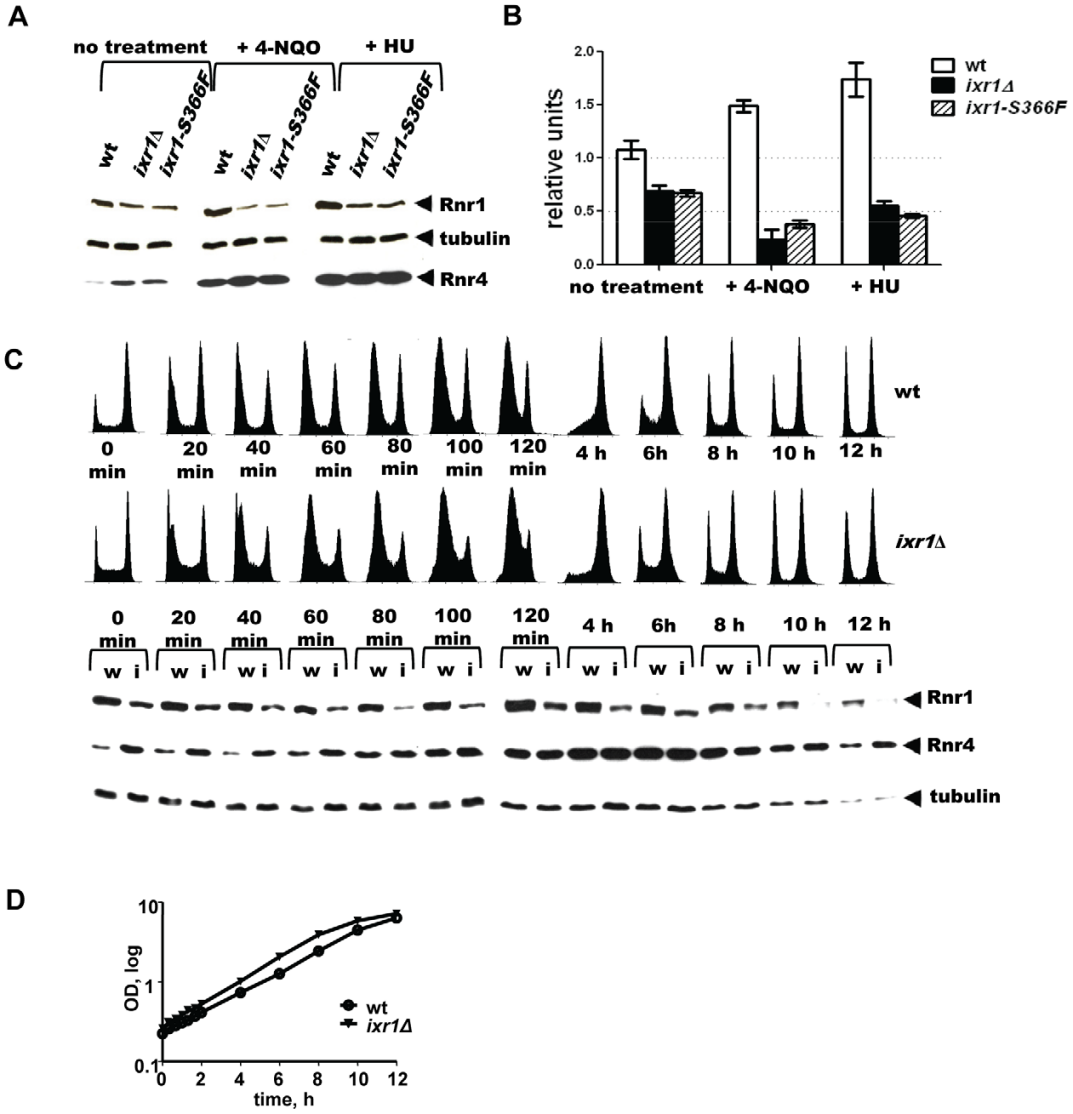
Rnr1 levels are reduced in *ixr1* after DNA damage. (A) Western blot analysis of Rnr1 levels in wild-type (wt) (W1588-4C), *ixr1-S366F* (TOY734), and *ixr1*Δ (TOY736) strains before and after 2 hours treatment with 0.2 mg/L 4-NQO or 200 mM HU. (B) Quantification of Rnr1 levels in wild-type (wt) (W1588-4C), *ixr1*Δ (TOY736) and *ixr1-S366F* (TOY734) strains before and after 2 hours treatment with 0.2 mg/L 4-NQO or 200 mM HU. Tubulin was used as the internal control (see Materials and methods). Error bars represent standard error of the mean (SEM). (C) Lower panel, dynamics of Rnr1 decrease and Rnr4 increase in wt (W1588-4C, lanes marked with “w”) and *ixr1*Δ (TOY736, lanes marked with “i”) strains during a 12-hour time-course incubation with 0.2 mg/L 4-NQO. Upper panel, the corresponding flow-cytometric histograms. (D) Proliferation curves for the experiment in Figure 4C.

The paradoxical finding that dNTP pools decreased despite the activation of the Mec1-Rad53-Dun1 pathway in *ixr1* strains indicates a deficiency in another component(s) of the RNR machinery. Analysis of Rnr1 steady state levels demonstrated moderately decreased levels in *ixr1*Δ and in *ixr1-S366F* compared to wild-type (∼64%, and ∼62% respectively) (Figure 5A, 5B, and 5C (0 min lanes)). As expected, incubation of wild-type cells in the presence of 4-NQO or HU for two hours led to an increase in Rnr1 levels (∼39% and 57%, respectively) (Figure 5A and 5B). Interestingly, the same treatment of *ixr1-S366F* and *ixr1*Δ led to a further reduction of Rnr1 to ∼19% and ∼37%, respectively, after 4-NQO treatment, and to ∼56% and ∼46%, respectively, after the HU treatment (Figure 5A and 5B). This reduction was continuous; Rnr1 levels remained lower in *ixr1*Δ throughout a 12-hour incubation with 4-NQO (Figure 5C). Based on flow-cytometric analysis, the *ixr1*Δ strain had a slightly greater proportion of cells in S phase compared to wild-type both before and during 4-NQO treatment (Figure 5C), although the overall proliferation rate was similar between *ixr1*Δ and wild-type (Figure 5D).

The decreased Rnr1 levels caused by *ixr1*Δ provide an explanation for the synthetic lethality displayed by the *ixr1*Δ *dun1*Δ double mutant strain: the *dun1*Δ strains are defective in relieving the inhibition of RNR imposed by Sml1, Dif1, and Crt1.

### Deletion of *IXR1* negatively affects *RNR1* transcription

**Figure 6:**
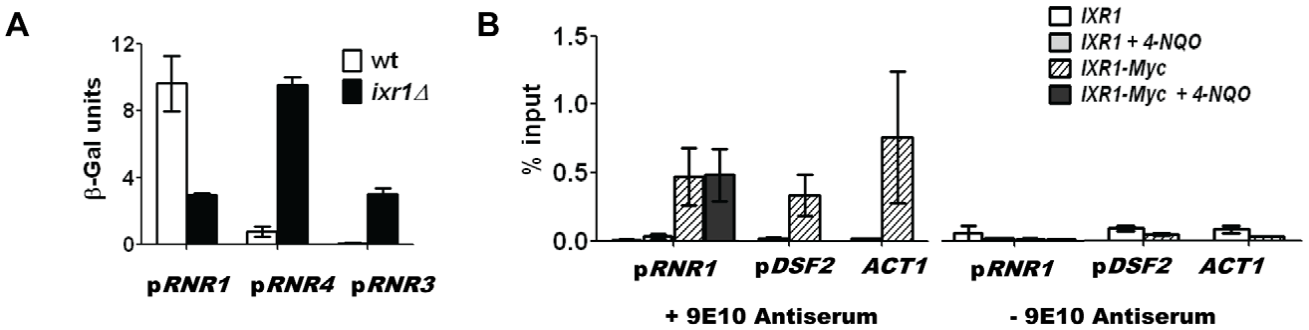
Ixr1 regulates *RNR1* promoter activity. (A) β-Galactosidase assay of *RNR1*, *RNR3*, and *RNR4* promoter activity in wild-type (W1588-4C) and *ixr1*Δ (TOY736) strains. Promoters of the analyzed genes were fused with the *lacZ* repoter gene and the respective plasmids were transformed in the wild type and *ixr1*Δ strains. β-Gal units were quantified as described in Materials and Methods. (B) Analysis of DNA associated with Ixr1 was performed by chromatin immunoprecipitation (ChIP) followed by qPCR using locus-specific primers for the *RNR1* promoter (p*RNR1*), the *DSF2* promoter (p*DSF2*) and the *ACT1* gene. ChIP was performed with *IXR1* (W1588-4C) and *IXR1-9MYC* (TOY836) strains using anti-Myc antiserum 9E10 or mock-antiserum. For *pRNR1*, ChIP was performed both without and with addition of 4-NQO (0.2 mg/L). Y axis represents the amount of precipitated DNA relative to the input DNA. Values shown are the average from two independent experiments with the minimum and maximum values represented.

Using a β-galactosidase assay we demonstrated that the decreased Rnr1 levels in *ixr1*Δ are due to a lower *RNR1* promoter activity, which indicates that Ixr1 directly or indirectly regulates *RNR1* transcription (Figure 6A). Deletion of *IXR1* also caused a concomitant increase in *RNR3* and *RNR4* promoter activities (Figure 6A), in agreement with the observed increase in Rnr3 and Rnr4 protein levels (Figure 3A). To gain further insight into the mechanism of *RNR1* regulation by Ixr1, we performed chromatin immunoprecipitation (ChIP) experiments followed by qPCR using Ixr1-9xMyc fusion protein and 9E10 antiserum. We analyzed binding of Ixr1 to the *RNR1* promoter (p*RNR1*) region. In addition, we analyzed the *DSF2* promoter (p*DSF2*) region earlier identified as an Ixr1-interacting locus [27] and the actin (*ACT1*) open reading frame, a commonly used negative control. As another control, we used an untagged congenic *IXR1* strain. Ixr1 interacted with all three loci (Figure 6B): relative to the input DNA we recovered 0.46%, 0.35% and 0.79% of p*RNR1*, p*DSF2* and *ACT1* loci, respectively, in the *IXR1*-9Myc strain. The interaction of Ixr1 with the *RNR1* promoter did not change after the treatment of cells with 4-NQO (0.46% and 0.48%, respectively). In the untagged strain, DNA recovery was at background levels as judged by the ChIP samples where 9E10 antiserum was omitted. The precipitation of the *ACT1* ORF locus indicates that Ixr1 binds to many loci in the genome.

### Elevation of Rnr1 levels in response to DNA damage depends on Rad53, but not Dun1

**Figure 7:**
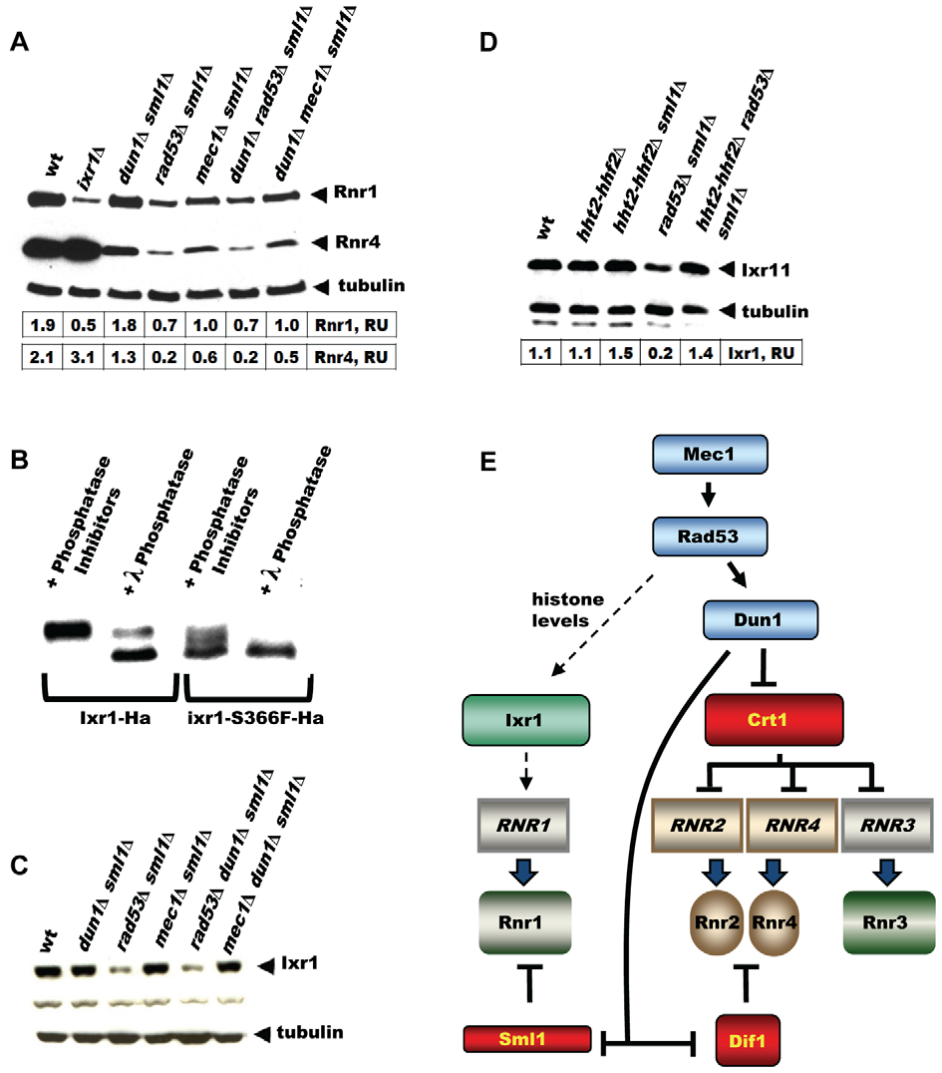
Elevation of Rnr1 levels in response to DNA damage requires *MEC1*, *RAD53*, and *IXR1*, but not *DUN1*. (A) Western blot analysis of Rnr1 and Rnr4 levels. The following strains were incubated with 0.2 mg/L 4-NQO for 2 hours: wt (W1588-4C), *ixr1*Δ (TOY736), *dun1*Δ *sml1*Δ (TOY728), *rad53*Δ *sml1*Δ (TOY782), *mec1*Δ *sml1*Δ (TOY711), *dun1*Δ *rad53*Δ *sml1*Δ (TOY786), and *dun1*Δ *mec1*Δ *sml1*Δ (TOY774). Rnr1 and Rnr4 levels were quantified in relative units (RU, levels of Rnr1 or Rnr4 divided by the levels of tubulin in corresponding sample) as described in Materials and Methods. (B) Western blot analysis of λ-phosphatase treated extracts from Ixr1-Ha (TOY655) and Ixr1-S366F-Ha (TOY650) strains. (C) Western blot analysis of Ixr1 levels in wt (W1588-4C), *dun1*Δ *sml1*Δ (TOY728), *rad53*Δ *sml1*Δ (TOY782), *mec1*Δ *sml1*Δ (TOY711), *rad53*Δ *dun1*Δ *sml1*Δ (TOY786) and *mec1*Δ *dun1*Δ *sml1*Δ (TOY774). (D) Western blot analysis of Ixr1 levels in wild-type (W1588-4C), *hht2-hhf2*Δ (TOY806), *hht2-hhf2*Δ *sml1*Δ (TOY821), *rad53*Δ *sml1*Δ (TOY782) and *hht2-hhf2*Δ *rad53*Δ *sml1*Δ (TOY819). Ixr1 levels were quantified in relative units (RU, levels of Ixr1 divided by the levels of tubulin in corresponding sample) as described in Materials and Methods. (E) Mec1-Rad53-Dun1-dependent regulation of *S. cerevisiae* ribonucleotide reductase. Expression of *RNR1* depends on Mec1, Rad53 and Ixr1, but does not depend on Dun1 or Crt1.

The DNA-damage-inducible genes become damage uninducible in *dun1* mutants, because Dun1 is required to relieve the inhibition imposed by the transcriptional inhibitor Crt1. Indeed, induction of Rnr4 in response to 4-NQO is less pronounced in the *dun1*Δ *sml1*Δ strain compared to wt (Figure 7A). The *RNR1* promoter, however, does not contain Crt1 sites and the expression of *RNR1* is not affected by the *CRT1* deletion [17], [18]. In Figure 7A, we demonstrate that the elevation of Rnr1 levels in response to DNA damage does not depend on *DUN1*, but does depend on Rad53 and Mec1. All checkpoint mutant strains in this experiment contained *sml1*Δ, because *mec1*Δ and *rad53*Δ are inviable otherwise. As a control, we demonstrate that the deletion of *SML1* by itself has little effect on Rnr1 and Rnr4 levels in the wild type and *ixr1*Δ strains treated by 4-NQO (Figure S1C).

### Ixr1 levels depend on histone dosage

Because Mec1, Rad53 and Dun1 are protein kinases, it was possible that they directly phosphorylate Ixr1 and modulate its function. We analyzed the phosphorylation status of Ixr1 and Ixr1-S366F proteins and found that Ixr1 is a phosphoprotein most likely phosphorylated at several residues (Figure 7B). Serine 366 is important for Ixr1 phosphorylation, as Ixr1-S366F separated by SDS-PAGE as several bands with higher mobility compared to Ixr1. Treatment of Ixr1 with λ-phosphatase increased the mobility of Ixr1 to that of Ixr1-S366F. However, we did not observe changes in Ixr1 mobility in *mec1*, *rad53*, or *dun1* mutants (Figure 7C). Interestingly, Ixr1 levels were significantly lower in *rad53*Δ, but not in *mec1*Δ or *dun1*Δ, compared to wt (Figure 7C). Rad53 is known to regulate histone levels, and *rad53* strains have increased amount of histones [28], [29]. Lowering histone dosage in the *rad53* mutant strains by deleting copies of histone 3 and histone 4 genes (*HHT2* and *HHF2*) restored Ixr1 to wild-type levels (Figure 7D).

## Discussion

The Dun1 kinase is a downstream target of the Mec1-Rad53 checkpoint, which monitors the genome integrity. In *S. cerevisiae*, the Mec1-Rad53-Dun1 pathway also regulates the RNR activity both during the normal cell cycle and after DNA damage [4]. RNRs are instrumental in controlling dNTP balance and concentration [30]. Deletion of *DUN1* is synthetic lethal with the deletion of many genes involved in DNA replication and DNA repair [20], [21]. Synthetic lethality of *dun1* with a number of other genes remains unexplained. Here, we demonstrate that *IXR1*, deletion of which is synthetic lethal with *dun1*, is required for the normal expression of the *RNR1* gene and maintenance of the dNTP pools.

In the absence of DNA damage, the deletion of *IXR1* leads to a moderate decrease of Rnr1 and dNTP levels. This decrease is partially compensated by the activation of the Mec1-Rad53-Dun1 pathway. We base this conclusion on the following observations. First, Rnr3 and Rnr4, whose levels are controlled by the Mec1-Rad53-Dun1 pathway, are upregulated in *ixr1*, and Rad53 is required for this upregulation. Second, RNR inhibitor Sml1, whose levels are also controlled by the Mec1-Rad53-Dun1 pathway, is downregulated in *ixr1*. Third, *DUN1* is indispensable in *ixr1*, but *ixr1 dun1*Δ synthetic lethality is rescued by elevated dNTP levels. We note that elevation of dNTP levels in *ixr1*Δ caused by the *SML1* deletion does not eliminate the checkpoint activation (Figure 3E), indicating that deletion of *IXR1* leads to replication stress not only because of the decreased dNTP levels expression but also because of other defects. It is conceivable that in addition to *RNR1* some other genes involved in the DNA biosynthesis are regulated by Ixr1. The reported synthetic lethality of *ixr1*Δ with the origin recognition complex mutant *orc2-1* [31] and synthetic sickness with the thymidylate kinase mutant *cdc8-2* [32] indicate the importance of Ixr1 for the processes involved in DNA replication.

RNR expression increases in response to DNA damage in most organisms. In *E. coli*, *nrdA* and *nrdB* (encoding the large and the small RNR subunits, respectively) are among the most highly induced genes following UV exposure (induced ∼20- and ∼7-fold, respectively) [33], [34]. In mammalian cells, DNA damage induces the p53R2 protein, an alternative small RNR subunit, about 4-fold in a p53-dependent manner [35]–[37]. Similarly, the *Drosophila* large RNR subunit, RnrL, is induced by ionizing radiation in wild-type, but not p53-deficient strains [38]. In the yeast *Schizosaccharomyces pombe*, *RNR* genes are among the most robustly induced genes following DNA damage [39]. All four *S. cerevisiae RNR* genes are activated by DNA damage and replication blocks [8], [10], [12]. The pathway involved in the activation of *RNR2*, *RNR3* and *RNR4* is well understood and requires the Mec1-Rad53-Dun1 kinase cascade, which targets Crt1, transcriptional inhibitor of DNA-damage-inducible genes (Figure 1A). Here we demonstrate that elevation of Rnr1 in response to DNA damage requires Mec1 and Rad53, but not Dun1 (Figure 7A). Earlier, it has been shown that *RNR1* expression does not depend on Crt1 [17], [18]. Thus, the downstream Dun1-Crt1 part of the Mec1-Rad53-Dun1-Crt1 pathway, which is known to control the DNA-damage-inducible genes in yeast, is not involved in the regulation of Rnr1. Instead, the elevation of Rnr1 levels in response to DNA damage requires Ixr1 (Figure 7E).

In addition to Crt1, transcription of *RNR2*, *RNR3* and *RNR4* genes is also controlled by Rox1 and Mot3, the DNA binding proteins that repress the hypoxic genes by recruiting the Ssn6/Tup1 general repression complex. Again, in contrast to *RNR2*, *RNR3*, and *RNR4* genes, no Rox1 or Mot3 sites are present in the *RNR1* promoter [18]. Transcription of *RNR1* is controlled by MBF, a dimeric transcription factor composed of Swi6 and Mbp1 [40]–[42]. Interestingly, Swi6 is directly phosphorylated by Rad53 in response to DNA damage [43]. It will be interesting to investigate whether Ixr1 is important for MBF-dependent regulation of the *RNR1* promoter. Earlier, Ixr1 was implicated in controlling the levels of the hypoxic gene *COX5b* [24]. Currently, we do not know whether Ixr1 is involved in the activation of *RNR1* expression in response to oxygen deprivation.

In contrast to many other HMG-box proteins, Ixr1 is rather large (68 kDa) and contains several polyglutamine repeats, which are often involved in protein-protein interactions and are present in many transcription factors. The HMG box is a conserved domain of ∼80 amino acids, binding to the minor groove of DNA. Proteins containing two or more HMG boxes usually recognize structural features of DNA without sequence specificity, while proteins containing one HMG box can recognize DNA in a sequence specific manner. In *S. cerevisiae*, there are two proteins containing two HMG boxes (Ixr1 and Abf2) and five proteins containing one HMG box (Nhp6A, Nhp6B, Nhp10, Hmo1 and Rox1). The closest homolog of Ixr1, Abf2, binds to many loci in the mitochondrial genome [44]. Yet, the HMG-box proteins with two or more HMG boxes can bind to specific loci in the genome. For example, human transcription factor UBF, which has 6 HMG boxes and belongs to the sequence-nonspecific class of HMG-box proteins, binds specifically to rDNA or to heterologous UBF-binding sequences from *Xenopus* integrated into ectopic sites on human chromosomes [45]. Our ChIP analysis of Ixr1 identified the *RNR1* promoter as a binding locus. However, Ixr1 bound equally well at two other tested loci, the *DSF2* promoter and the *ACT1* open reading frame. Still, it is possible that, in the context of the *RNR1* promoter, Ixr1 together with other proteins directly regulates *RNR1* gene expression.

Interestingly, the mutation in the Ixr1 S366 residue that is important for interaction of HMG boxes with DNA results in the same phenotype as the deletion of *IXR1* gene. We show that this serine residue is required for the phosphorylation of several amino acid residues in Ixr1. Currently we do not know the phosphorylation status of S366. It is possible that S366 itself is not phosphorylated, but its interaction with the DNA or other proteins is required for the phosphorylation of other residues in Ixr1. Multiple phosphorylation of Ixr1 causes an increase in the apparent molecular weight of the protein: Ixr1 separates by SDS-PAGE as a ∼85 kDa protein (not as predicted 68 kDa). We demonstrate that neither Mec1, nor Rad53, nor Dun1 are responsible for the phosphorylation of Ixr1, as its mobility is not affected in the respective mutants. The region of the HMG domain around the Ser366 residue has been shown to affect DNA binding specificity. All sequence-specific HMG proteins have an asparagine at this position, whereas all non-sequence-specific HMG proteins have a serine (e.g., Ser10 in the *D. melanogaster* HMG-D box co-crystallized with DNA) [26]. To our knowledge, crystal structures analyzing the interaction of HMG boxes and DNA were solved with the non-phosphorylated proteins. It would be interesting to investigate whether S366 is phosphorylated in Ixr1, whether the corresponding serine residues are phosphorylated in other HMG proteins in other species, and whether Ser366 phosphorylation affects DNA binding and/or makes binding of the HMG box sequence specific.

Although the mobility of Ixr1 is not changed, its levels are significantly reduced in the *rad53*Δ strain (Figure 7C). *rad53* mutant strains are known to have increased histone levels due to a defect in histone degradation [28]. Increased histone levels presumably lead to decreased Ixr1 levels, because we show that decreasing histone dosage in the *rad53* strain restores Ixr1 levels (Figure 7D). There are at least two possibilities explaining this interplay between Ixr1 and histone levels. Because Ixr1 contains two HMG boxes and therefore binds DNA presumably without sequence specificity, it might compete with histones for DNA binding. Increased histones in *rad53*Δ might displace Ixr1 from the *IXR1* promoter, where it was shown to bind and regulate its own expression [46]. Alternative, but not exclusive possibility is that Ixr1 displaced by histones from DNA undergoes degradation.

In summary, we identify Ixr1 as a novel factor involved in regulation of dNTP pools and *RNR1*, a gene that, in contrast to all other known DNA-damage inducible genes, is not controlled by Dun1 and Crt1.

## Materials and Methods

### Yeast strains and primers

All yeast strains used in this study are congenic to W1588-4C [5]. Table 1 gives only the allele(s) that differ from the W1588-4C genotype. Table S1 lists primers used for strain construction. *DUN1* was deleted using the KanMX4 cassette PCR-amplified with primers F_Dun1 and R_Dun1 from the *dun1*Δ*::KanMX4* Y03798 strain (Euroscarf). The CY1263 *ade3::HISG* strain [47] was crossed with W1588-4C to select *ade2 ade3* clones (TOY502). The resulting strain was crossed with *dun1Δ::KanMX4* to create the strain used for the synthetic lethality screen (TOY527). The TOY836 (*IXR1-9MYC*) strain was generated by amplifying and introducing the *9MYC-TRP1* cassette from the Z1580 strain [48] into W1588-4C.

**Table 1:** Yeast strains used in this study.

Strain	Genotype	Reference
W1588-4C	*MAT* **a** *ade2-1can1-100 his3-11.15 leu2-3,112 trp1-1ura3-1 RAD5^+^*	[5]
AC447-2A	*MAT* **a** *ade2-1 can1-100 his3-11,15 leu2-3,112 trp1-1 ura3-1 rad5 RNR3-3HA::KanMX6*	[49]
U952-3B	*MAT* **a** *sml1*Δ*::HIS3*	[5]
CY1263	*MAT*α *ade3::HISG*	[47]
CUY995	*MAT*α *rnr4-D1::HIS3 ade2-101 lue2-3,112 ura3-52*	[11]
TOY502	*MAT* **a** *ade3::HISG*	This study
TOY510	MAT**a** *dun1*Δ*::KanMX6*	This study
TOY527	*MAT*α *ade3::HISG dun1*Δ*::KanMX6*	This study
TOY541	*MAT*α *ade3::HISG dun1*Δ*::KanMX6* [pK503] (*ura3*Δ*::LEU2, ADE3, DUN1*)	This study
TOY544	*MAT* **a** *ade3::HISG dun1*Δ*::KanMX6 ixr1-S633F* [pK503]	This study
TOY566	*MAT* **a** *ade3::HISG dun1*Δ*::URA3*	This study
TOY588	MAT**a** *ade3::HISG dun1*Δ*::KanMX6 sml1*Δ*::HIS3*	This study
TOY598	*MAT*α *ade3::HISG dun1*Δ*::KanMX6 ixr1-S366F::TRP1* [pK503]	This study
TOY603	*MAT* **a** *ade3::HISG ixr1*Δ*::TRP1*	This study
TOY604	*MAT* **a** *ade3::HISG ixr1*Δ*::TRP1 sml1*Δ*::HIS3*	This study
TOY619	*MAT* **a** *ade3::HISG ixr1-S366F::TRP1 RNR3-Ha::KanMX6*	This study
TOY621	*MAT* **a** *ade3::HISG ixr1*Δ*::TRP1 RNR3-Ha::KanMX6*	This study
TOY650	*MAT* **a** *ade3::HISG ixr1-S366F-Ha::KanMX6*	This study
TOY655	*MAT* **a** *ade3::HISG IXR1-Ha::KanMX6*	This study
TOY711	MAT**a** *mec1*Δ*::TRP1 sml1*Δ*::HIS3*	This study
TOY714	*MAT* **a** *ixr1*Δ*::trp1*Δ*::URA3 sml1*Δ*::HIS3*	This study
TOY728	MAT**a** *dun1*Δ*::KanMX6 sml1*Δ*::HIS3*	This study
TOY732	*MAT* **a** *ixr1*Δ*::TRP1 RNR3-Ha::KanMX6*	This study
TOY734	*MAT* **a** *ixr1-S366F::TRP1*	This study
TOY736	*MAT* **a** *ixr1*Δ*::TRP1*	This study
TOY772	*MAT* **a** *ixr1*Δ*::trp1*Δ*::URA3 dun1*Δ*::ura3*Δ*::LEU2 sml1*Δ*::HIS3 RNR3-Ha::KanMX6*	This study
TOY774	*MAT* **a** *dun1*Δ*::ura3*Δ*::LEU2 mec1*Δ*::TRP1 sml1*Δ*::HIS3 RNR3-Ha::KanMX6*	This study
TOY778	*MAT* **a** *ixr1*Δ*::trp1*Δ*::URA3 sml1*Δ*::HIS3 RNR3-Ha::KanMX6*	This study
TOY781	*MAT* **a** *ixr1*Δ*::trp1*Δ*::URA3 rad53*Δ*::HphMX4 sml1*Δ*::HIS3 RNR3-Ha::KanMX6*	This study
TOY782	*MAT* **a** *rad53*Δ*::HphMX4 sml1*Δ*::HIS3 RNR3-Ha::KanMX6*	This study
TOY786	*MAT* **a** *dun1*Δ*::ura3*Δ*::LEU2 rad53*Δ*::HphMX4 sml1*Δ*::HIS3 RNR3-Ha::KanMX6*	This study
TOY806	*MAT* **a** *hht2-hhf2*Δ*::KanMX6*	This study
TOY819	*MAT* **a** *rad53*Δ*::HphMX4 hht2-hhf2*Δ*::KanMX6 sml1*Δ*::HIS3*	This study
TOY821	*MAT* **a** *hht2-hhf2*Δ*::KanMX6 sml1*Δ*::HIS3*	This study
TOY836	*MAT* **a** *IXR1-9Myc::TRP1*	This study

### Plasmids

To overexpress Rnr1 or Rnr3, the previously described pESC-pGAL1-*RNR1* or pBAD79 plasmids were used [6], [49]. To express *RNR1* under its own promoter from a low-copy centromeric vector, the pBJ6 (pRS316-*RNR1*) plasmid was used (gift of Anders Byström, Umeå University). To construct pK503 (*ura3Δ::LEU2*, *ADE3*, *DUN1*), the *DUN1* gene including the promoter region was PCR-amplified using primers Dun1_F and Dun1_rev. The PCR product was cloned into the *Sal*I site of p2013 [50], and the *URA3* gene in the resulting plasmid was then replaced by *LEU2*. To construct pK521 (*TRP1*, *DUN1*) a *Sal*I/*Sal*I fragment of *DUN1* from pK503 was cloned into the *Sal*I site of pRS414 [51]. To construct plasmids for the β-Galactosidase assay, the *RNR1*, *RNR3* and *RNR4* promoters were PCR amplified from the W1588-4C genomic DNA using primers pR1-F, pR1-R, pR3-F, pR3-R, pR4-F and pR4-R. The *RNR3* promoter was cloned in the *Bam*HI site of pJO20, and the *RNR1* and *RNR4* promoters were cloned in the *Bam*HI site of pJO21 [52], resulting in plasmids pK505, pK504 and pK506, respectively. β-Galactosidase levels were assayed as described [52].

### Synthetic lethal screen

To identify mutations synthetic lethal with *dun1*Δ, we used a color-based synthetic lethal screen [53]. The TOY541 strain carrying pK503 was grown in selective medium to ∼2×10^7^ cells/ml. Cells were spun down, resuspended in water, plated onto YPD plates at 1500 cells/plate, and UV-mutagenized with a dose of 150 J/m^2^, resulting in 30% survival. Plates were placed in the dark and incubated for 3 days at 30°C. Non-sectoring red colonies were re-streaked twice on YPD, and those retaining the red color were selected for further analysis. Candidate mutants were transformed with pK521 to exclude mutants synthetically lethal with the plasmid-borne *ADE3* or *LEU2* genes. Transformants were grown on –Trp medium, and strains with a sectoring phenotype were selected. The candidate mutants were crossed with TOY566 to test recessiveness/dominance, and tetrad analysis was performed to select mutations with monogenic inheritance. Selected strains were mated in all possible combinations to establish complementation groups. In total, we isolated 4 mutants falling into three complementation groups and identified them as one *ixr1*, one *rad53* and two *whi3* mutants as outlined below.

One strain from each complementation group was transformed with a pRS314-based yeast genomic DNA library [54], and transformants were selected on –Trp plates. Clones that showed a sectoring phenotype were re-streaked onto –Trp plates, and plasmids were isolated from these clones and partially sequenced using T3 and T7 standard primers. The obtained sequences were subjected to BLAST homology searches using the *S. cerevisiae* genome database, and genomic regions were retrieved. One of the regions contained the *IXR1* ORF (TOY544). A *TRP1* cassette was inserted downstream of the *IXR1* ORF creating TOY598, which was crossed with TOY566 to verify the co-segregation of a genomic marker and the non-sectoring phenotype. Then, the genomic region retrieved from the DNA library in pRS314 was shortened, and resulting plasmids were re-transformed in TOY544. Plasmids lacking the full-length *IXR1* ORF failed to recover the sectoring phenotype. *IXR1* and *ixr1* ORFs were PCR amplified from the genomic DNA of TOY502 and TOY544, respectively, using primers F_ixr and R_ixr, and sequenced using the same primers. The mutations in *RAD53* and *WHI3* genes were identified by the same procedure and PCR amplified followed by sequencing using primers F_rad53, R_rad53, F_whi3 and R_whi3.

### Western blotting and antibodies

Protein samples for Western blotting were prepared as described [55]. Proteins were separated by SDS-PAGE and transferred to a nitrocellulose membrane (Protran BA 85, Whatman, USA) using the Minigel System (C.B.S. Scientific Co., USA).

Rabbit polyclonal anti-Rnr1 (AS09 576), anti-Rnr2 (AS09 575), anti-Rnr3 (AS09 574), and anti-Sml1 (AS10 847) antibodies were produced by Agrisera, Sweden (peptides used for immunization are listed in Table S2). For the detection of Ixr1 we used rabbit polyclonal antibodies produced by Agrisera, Sweden (Table S2). For the detection of the HA-tag, mouse monoclonal 12CA5 antibodies were used (1∶5000). For the detection of both Rnr4 and α-tubulin [56], we used YL1/2 rat monoclonal antibodies (Sigma) at 1∶2500. These antibodies recognize C-termini of α-tubulin and small RNR subunits from different species. The absence of the Rnr4 band on a Western blot with an extract from an *rnr4*Δ strain (CUY995, [11]) confirmed that YL1/2 antibodies specifically recognize yeast Rnr4 (Figure S1B). For the detection of Rad53, we used yC-19 goat polyclonal antibodies at 1∶2000 (Santa Cruz Biotechnology, USA).

Quantification of protein levels was performed using ImageJ software (http://rsbweb.nih.gov/ij). Protein levels were calculated as relative units (RU, levels of the particular protein divided by the levels of tubulin in corresponding sample). To quantify Rnr1 levels three independent clones were analyzed on the same membrane.

### Chromatin immunoprecipitation and quantative PCR

Chromatin immunoprecipitation followed by qPCR was performed as previously described (Barsoum et al., 2010). DNA damaging agent 4-NQO was added to the cells to final concentration 0.25 mg/L at OD ∼0.5 and cells were grown 2 hours to OD ∼1.2–1.5. To amplify *RNR1* promoter, *DSF2* promoter and *ACT1* open reading frame ChIP_pRNR1, ChIP_pDSF2 and ChIP_ACT1 primers were used (Table S1).

### Treatment with λ Phosphatase

9×10^7^ cells were collected, vortexed with glass beads in 10% w/v trichloroacetic acid and spun down 10 min in microcentrifuge in cold room. Pellet was re-suspended in 150 µl of λ-Phosphatase buffer, pH was adjusted to 7.5 with basic 1 M Tris and 15 µl of 10× Complete Protease Inhibitor Cocktail (Roche Applied Biosystems) was added to the samples. 60 µl of 10× PhosStop Phosphatase Inhibitor Cocktail (Roche Applied Biosystems) or 6 µl of λ Phosphatase (New England Biolabs) was added to the respective samples and all samples were incubated 1 hour at 30°C. Then, samples were boiled 10 min with Laemmli buffer and analyzed by SDS PAGE followed by the Western blotting.

### Measurement of dNTP levels

NTP and dNTP extraction and quantification were performed as previously described [7]. Nucleotides were analyzed by HPLC on a Partisphere SAX-5 HPLC column (4.6 mm×125 mm, Whatman International Ltd.) using a UV-2075 Plus detector (Jasco, Tokyo, Japan).

### Analysis of HU tolerance and measurement of the frequency of petite formation

Mid-log phase cells were collected, sonicated, and plated at appropriate dilutions. For spot assays, 2 µl of 10-fold serial dilutions were spotted onto YPD plates or YPD plates containing 200 mM HU. Cells were grown at 30°C for 3 days. Measurement of the frequency of petite formation was done as described before [5].

## Supporting Information

Figure S1 (A) Western blot analysis of Rnr2, Rnr3-HA (detected with anti-HA or with rabbit polyclonal anti-Rnr3 antibodies), and Rnr4 in the wild-type (wt) (AC447-2A) and in *ixr1*Δ (TOY621) strains before and after 2 hours treatment with 0.2 mg/L 4-NQO. (B) Specificity of the YL1/2 antibodies used for the detection of Rnr4. Wild-type (W1588-4C), *ixr1*Δ (TOY736), and *rnr4*Δ (CUY995) strains were analyzed before and after treatment with 0.2 mg/L 4-NQO. The Rnr4 band is absent in the *rnr4*Δ strain. Instead, a band of higher molecular weight appears in the position corresponding to Rnr2. (C) Western blot analysis of Rnr1 and Rnr4 levels in the wild-type (wt) (W1588-4C), *ixr1*Δ (TOY736), *sml1*Δ (U952-3B), and *ixr1*Δ *sml1*Δ (TOY778) strains after 2 hours treatment with 0.2 mg/L 4-NQO. Rnr1 and Rnr4 levels were quantified as described in Materials and Methods. RU, relative units.
(TIF)

Table S1 Primers used in this study. All insertions were confirmed with PCR, each strain was back-crossed with W1588-4C.
(DOCX)

Table S2 Antibodies used in this study.
(DOCX)
