# Supplementary figures and images for: Ixr1 Is Required for the Expression of the Ribonucleotide Reductase Rnr1 and Maintenance of dNTP Pools
Source: PLoS Genet. 2011 May 5;7(5):e1002061. doi: 10.1371/journal.pgen.1002061 (PMC3088718; doi:10.1371/journal.pgen.1002061)

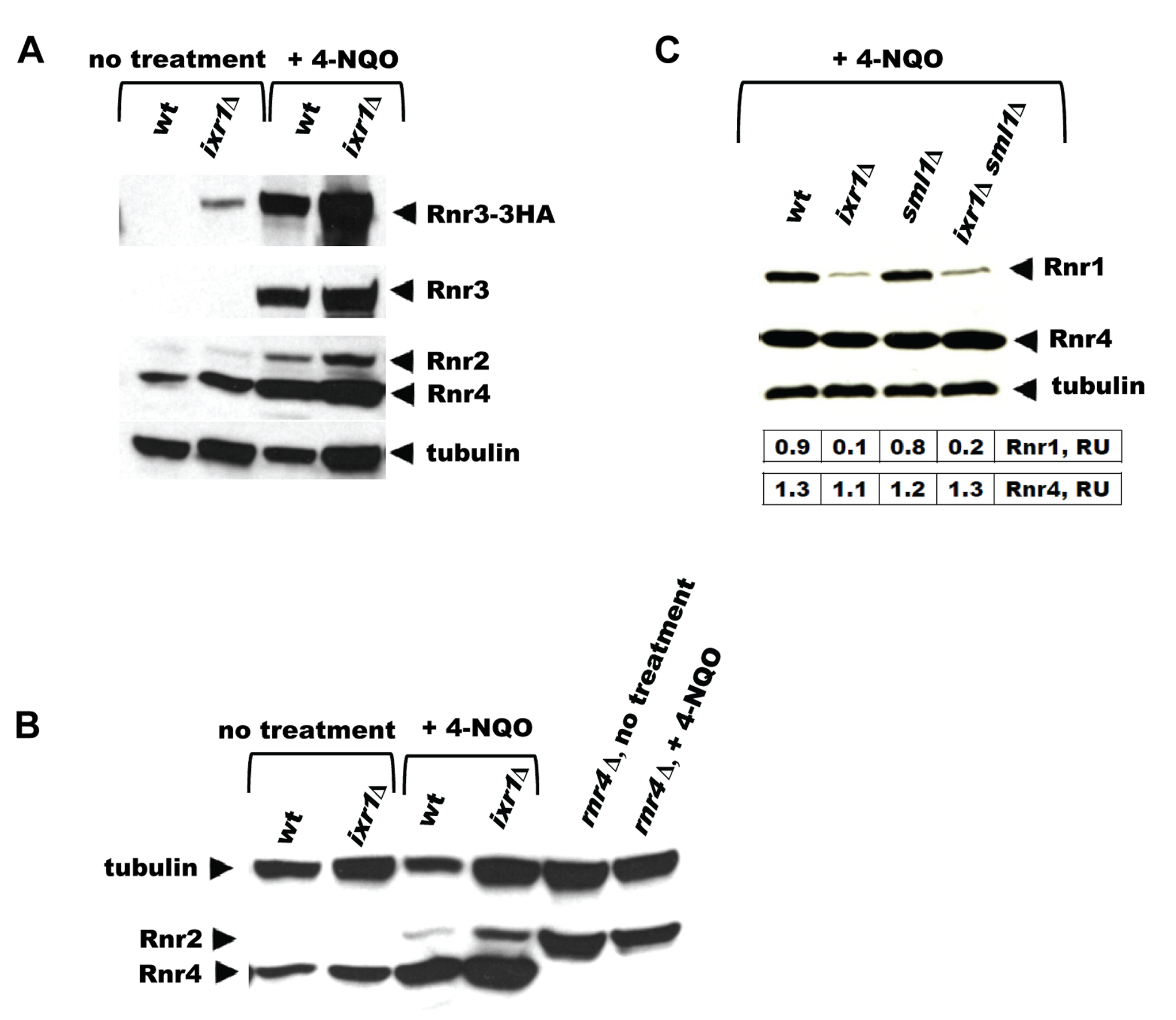

Supplement: Figure S1 — (A) Western blot analysis of Rnr2, Rnr3-HA (detected with anti-HA or with rabbit polyclonal anti-Rnr3 antibodies), and Rnr4 in the wild-type (wt) (AC447-2A) and in ixr1Δ (TOY621) strains before and after 2 hours treatment with 0.2 mg/L 4-NQO. (B) Specificity of the YL1/2 antibodies used for the detection of Rnr4. Wild-type (W1588-4C), ixr1Δ (TOY736), and rnr4Δ (CUY995) strains were analyzed before and after treatment with 0.2 mg/L 4-NQO. The Rnr4 band is absent in the rnr4Δ strain. Instead, a band of higher molecular weight appears in the position corresponding to Rnr2. (C) Western blot analysis of Rnr1 and Rnr4 levels in the wild-type (wt) (W1588-4C), ixr1Δ (TOY736), sml1Δ (U952-3B), and ixr1Δ sml1Δ (TOY778) strains after 2 hours treatment with 0.2 mg/L 4-NQO. Rnr1 and Rnr4 levels were quantified as described in Materials and Methods. RU, relative units. (TIF) [file pgen.1002061.s001.tif]
