# Supplementary material for: Ixr1 Is Required for the Expression of the Ribonucleotide Reductase Rnr1 and Maintenance of dNTP Pools
Source: PLoS Genet. 2011 May 5;7(5):e1002061. doi: 10.1371/journal.pgen.1002061 (PMC3088718; doi:10.1371/journal.pgen.1002061)
Supplement: Table S1 — Primers used in this study. All insertions were confirmed with PCR, each strain was back-crossed with W1588-4C. (DOCX) [file pgen.1002061.s002.docx]

Table S1

| Strain/ Primer | Sequence | Source | Reference |
| --- | --- | --- | --- |
| F_Dun1 | 5’ TAGAAGATAAGGAATAGAAGCCCCTG 3’ |  |  |
| R_Dun1 | 5’ GTTAAGGTTAAAAAAGAGCTG 3’ |  |  |
| Dun1_F | 5’ GATAAGGTCGACAAGCCCCTGAATACCATAAATAGAT 3’ |  |  |
| Dun1_R | 5’ TTTGAAGTCGACCTTCTCATGTTTAGAGGC 3’ |  |  |
| F_ixr | 5’ CAACGCAAGTCAAGGCAAGA 3’ |  |  |
| R_ixr | 5’ CTCGTTGTTTGCGTGGGATA 3’ |  |  |
| F_rad53 | 5’ GTCAATGAAACGTGCATCTATTAAC |  |  |
| R_rad53 | 5’ TGGTATCTACCATCTTCTCTCTT |  |  |
| F_whi3 | 5’ GCCTTTATCGATCAATATTTCA |  |  |
| R_whi3 | 5’ GATACATGCAAGGAAATCAG |  |  |
| pR1-F | 5’ CGCCCTAGGATCACTGTGTATAGTAGATC 3’ |  |  |
| pR1-R | 5’ CGCGGATCCGGTCATGATGTTAATATATCAACAAATAAAG 3’ |  |  |
| pR3-F | 5’ CGCCCTAGGACAAGCACATAAAAAATCAG 3’ |  |  |
| pR3-R | 5’ ATAGGATCCGGTCATTTGTGTGGGAGTATTTGAT 3’ |  |  |
| pR4-F | 5’ ATACCTAGGGGTTGTTTGGCCGAGCGGT 3’ |  |  |
| pR4-R | 5’ ATAGGATCCGGTCATTGTAATAACTAATTGTGTGCGTTGC 3’ |  |  |
| TOY598 | F 5’ TGCATTTTTTTTTTGTTCCCATTCGTTCTCTCACCAAGGGGAGGTGGAAAAGAAAAGAAGCGGATCCCCGGGTTAATTAA 3’  R 5’ GACGGCCGCGCGGGGTTTTGTGTTGGGTGTGATGTCCGGATGCAACAGCAGCAAAGGAGAGAATTCGAGCTCGTTTAAAC 3’ | pFA6a-TRP1 | (Longtine et al, 1998) |
| TOY604 | F 5’ ATCCCCTTCTTCTATCCATTCTGTGATATACGTACGACGCTAACAGTACCCACAACTGCACGGATCCCCGGGTTAATTAA 3’  R 5’ TTATTCATTTTTTATGATCGAACCATTTGTAGTGGGGTCAGCCAATAACATGTGTTGTTGGAATTCGAGCTCGTTTAAAC 3’ | pFA6a-TRP1 | (Longtine et al, 1998) |
| TOY734 | F 5’ TGCATTTTTTTTTTGTTCCCATTCGTTCTCTCACCAAGGGGAGGTGGAAAAGAAAAGAAGCGGATCCCCGGGTTAATTAA 3’  R 5’ GACGGCCGCGCGGGGTTTTGTGTTGGGTGTGATGTCCGGATGCAACAGCAGCAAAGGAGAGAATTCGAGCTCGTTTAAAC 3’ | pFA6a-TRP1 | (Longtine et al, 1998) |
| TOY781 | F 5’ AGCTTTAAAAGAGAGAATAGTGAGAAAAGATAGTGTTACACAACATCAACTAAAACACATACGATTTAGGTGACAC 3’  R 5’ ctaccatcttctctcttaaaaaggggcagcattttctatgggtatttgtccttggAATACGACTCACTATAGGGAG 3’ | pAG32 | (Goldstein & McCusker, 1999) |
| TOY806 | F’ TGTTTGTATGATGTCCCCCCAGTCTAAATGCATAGAAAAAAAAAAATTCCCGCTTTATATCGGATCCCCGGGTTAATTAA  R 5’ ATGATCGTTATTTTATTTTTTGAAAGGCATGAAAATAATTTCAAACACCGATTGTTTAACCACCGATTGTGAATTCGAGCTCGTTTAAAC | pFA6a-KanMX6 | (Longtine et al, 1998) |
| TOY836 | F 5’ CAACGCAAGTCAAGGCAAGA  R 5’ CTCGTTGTTTGCGTGGGATA | Z1580 | (Lee et al, 2002) |
| ChIP_pRNR1 | F 5’ GGAATTCCATTGGGGAAAAT  R 5’ CGTTCCAGGGCTACGAAATA |  |  |
| ChIP_pDSF2 | F 5’ TTGCACCGTTGTTCTTTCTG  R 5’ GTGTGTTTCTGCGCGTTAAA |  |  |
| ChIP_ACT1 | 5’ TTGGCCGGTAGAGATTTGAC  5’ TCTGGGGCTCTGAATCTTTC |  |  |

References:

Goldstein AL, McCusker JH (1999) Three new dominant drug resistance cassettes for gene disruption in Saccharomyces cerevisiae. *Yeast* **15**(14)**:** 1541-1553

Lee TI, Rinaldi NJ, Robert F, Odom DT, Bar-Joseph Z, Gerber GK, Hannett NM, Harbison CT, Thompson CM, Simon I, Zeitlinger J, Jennings EG, Murray HL, Gordon DB, Ren B, Wyrick JJ, Tagne JB, Volkert TL, Fraenkel E, Gifford DK, Young RA (2002) Transcriptional regulatory networks in Saccharomyces cerevisiae. *Science* **298**(5594)**:** 799-804

Longtine MS, McKenzie A, Demarini DJ, Shah NG, Wach A, Brachat A, Philippsen P, Pringle JR (1998) Additional modules for versatile and economical PCR-based gene deletion and modification in Saccharomyces cerevisiae. *Yeast* **14**(10)**:** 953-961
