# Supplementary material for: Ixr1 Is Required for the Expression of the Ribonucleotide Reductase Rnr1 and Maintenance of dNTP Pools
Source: PLoS Genet. 2011 May 5;7(5):e1002061. doi: 10.1371/journal.pgen.1002061 (PMC3088718; doi:10.1371/journal.pgen.1002061)
Supplement: Table S2 — Antibodies used in this study. (DOCX) [file pgen.1002061.s003.docx]

Table S2

Antibodies used in this study

| Name | Antigen | Dilution used |
| --- | --- | --- |
| α-Rnr1 (762-773)  α-Rnr1 (825-838) | (NH_2_)-CLTKGMAELNVQESK-(CONH_2_)  (NH_2_)-CDQATENVADISN-(CONH_2_) | 1:5000 |
| α-Rnr3 (762-775) | (NH_2_)- DQAATHIASVSELD-(CONH_2_) | 1:5000 |
| α-Rnr2 (32-45)  α-Rnr2 (386-399) | (NH_2_)- CTLREENRVKSDMLK-(CONH_2_)  (NH_2_)- CTKQEAGAFTFNEDF-(CONH_2_) | 1:50000 |
| α-Ixr1 (2-14)  α-Ixr1 (502-515) | (NH_2_)- CNTGISPKQDDASN-(CONH_2_)  (NH_2_)- CNDPNGNPTGHSHKA-(CONH_2_) | 1:1000 |
| α-Sml1 | whole protein | 1:5000 |
